# Mice lacking cyclophilin B, but not cyclophilin A, are protected from the development of NASH in a diet and chemical-induced model

**DOI:** 10.1371/journal.pone.0298211

**Published:** 2024-03-01

**Authors:** Winston T. Stauffer, Asha Z. Goodman, Michael Bobardt, Daren R. Ure, Robert T. Foster, Philippe Gallay

**Affiliations:** 1 Department of Immunology & Microbiology, Scripps Research, La Jolla, California, United States of America; 2 Hepion Pharmaceuticals, Edison, New Jersey, United States of America; University of Rajshahi, BANGLADESH

## Abstract

Cyclophilins are a diverse family of peptidyl-prolyl isomerases (PPIases) of importance in a variety of essential cellular functions. We previously reported that the pan-cyclophilin inhibitor drug reconfilstat (CRV431) decreased disease in mice under the western-diet and carbon tetrachloride (CCl_4_) non-alcoholic steatohepatitis (NASH) model. CRV431 inhibits several cyclophilin isoforms, among which cyclophilin A (CypA) and B (CypB) are the most abundant. It is not known whether simultaneous inhibition of multiple cyclophilin family members is necessary for the observed therapeutic effects or if loss-of-function of one is sufficient. Identifying the responsible isoform(s) would enable future fine-tuning of drug treatments. Features of human liver fibrosis and complete NASH can be reliably replicated in mice by administration of intraperitoneal CCl_4_ alone or CCl_4_ in conjunction with high sugar, high cholesterol western diet, respectively. Here we show that while wild-type (WT) and Ppia-/- CypA KO mice develop severe NASH disease features under these models, Ppib-/- CypB KO mice do not, as measured by analysis of picrosirius red and hematoxylin & eosin-stained liver sections and TNFα immuno-stained liver sections. Cyclophilin inhibition is a promising and novel avenue of treatment for diet-induced NASH. In this study, mice without CypB, but not mice without CypA, were significantly protected from the development of the characteristic features of NASH. These data suggest that CypB is necessary for NASH disease progression. Further investigation is necessary to determine whether the specific role of CypB in the endoplasmic reticulum secretory pathway is of significance to its effect on NASH development.

## Introduction

Non-alcoholic fatty liver disease (NAFLD) covers a wide spectrum of disorders resulting from excessive fat deposition in the liver in the absence of excessive alcohol consumption or viral infection. It is regarded as the hepatic manifestation of the wider Metabolic Syndrome (MetS), which features dyslipidemia, hypertension, and insulin resistance, and is associated with poor diet, lack of exercise, and obesity [1, 2]. In the liver, these factors contribute to the most basic feature of NAFLD, steatosis, or the deposition and accumulation of micro-vesicular lipid droplets in hepatocytes. From this asymptomatic beginning, the disease can progress to the more advanced non-alcoholic steatohepatitis (NASH), which features inflammation in addition to steatosis and may also show increasing amounts of fibrotic scarring. While the liver is a regenerative organ, extensive fibrosis and impaired liver function in advanced NASH can lead to cirrhosis or hepatocellular carcinoma, which are not reversible and may require liver transplant to avoid death. NAFLD has an increasingly high prevalence globally, with up to 30% of the population exhibiting some form of the disease. While preventative lifestyle changes are helpful in halting or reversing the progression of NAFLD/NASH, new drug treatments are necessary to accelerate or augment this process [3–5].

Previous studies have discovered that drugs which inhibit the activity of cyclophilins are beneficial in the treatment of fibrotic disease, including NASH [6–9]. Cyclophilins are a large family of peptidyl-prolyl isomerases (PPIases) ubiquitously present in all bodily tissues and cell types. All cyclophilin PPIases catalyze the *cis-trans* isomerization of peptide bonds between proline residues and any other amino acid [10, 11]. They are thus essential for the folding of nascent proteins into their final 3-dimensional forms. Cyclophilin family members, of which cyclophilin A (CypA, PPIA), cyclophilin B (CypB, PPIB), and cyclophilin D (CypD, PPIF) are the best characterized, have diverse localizations and functions within the cell and are known to be important players in many pathologies. CypA is a critical mediator of the inflammatory response [12, 13] and is also important in the lifecycles of many viruses, including hepatitis B and C [13–16]. CypB, is an effector of endoplasmic reticulum (ER) stress response and acts as a protein chaperone for a variety of proteins moving through the classical secretory pathway [17–19]. CypD forms part of the mitochondrial permeability pore and thus helps regulate cell death pathways [20]. The first cyclophilin inhibitor discovered was the immunosuppressant cyclosporin A (CsA), for which the cyclophilin family was named. CsA binds to CypA, creating a complex which inhibits calcineurin, a protein responsible for the activation of T-cells [21, 22]. More recent pan-cyclophilin inhibitors have been developed, including reconfilstat (CRV431) and NV556, which bind and disrupt cyclophilin activity but do not affect calcineurin, and thus have no effect on immune function [8, 23].

We have previously shown that CRV431, a CsA analog, and NV556, a derivative of sangliferhin A, another cyclophilin inhibitor, are noncytotoxic compounds that bind and inhibit all cyclophilin family members. Both have been shown to reduce fibrosis and the occurrence of HCC in multiple chronic liver disease models in mice, including those mimicking the features of human NAFLD/NASH [6, 7]. CRV431, in particular, was also shown to decrease fibrosis across multiple timepoints to a greater degree than the competitor compound, obeticholic acid (OCA). Given that CRV431 is a pan-cyclophilin inhibitor, it is unknown which cyclophilin family member(s) is(are) being acted upon to be mechanistically responsible for these results. In order to fine-tune future drug treatments, it is necessary to characterize which cyclophilin family member(s) play(s) the most important role in the progression of this disease.

In order to dissect which cyclophilin family member(s) is(are) most critical in the progression of NAFLD/NASH-related liver fibrosis, steatosis, and inflammation, we examined these disease models in two cyclophilin global knockout (KO) backgrounds. We found that, while CypA knockout mice were not significantly different from WT controls, CypB KO mice showed a marked and significant decrease in fibrotic staining, similar to non-diseased groups. Furthermore, CypB KO mice were alone in showing significantly blunted development of steatosis, inflammation, and other indicators of NAFLD/NASH.

## Materials and methods

### Laboratory animal use

The data presented here involving the use of laboratory animals were generated in accordance with the Institutional Animal Care and Use Committee (IACUC) of Scripps Research and conforms to the rules provided by the National Research Council’s Guide of the Care and Use of Laboratory Animals. Mice were monitored daily by Scripps Department of Animal Resources (DAR) staff and Dr. Stauffer who had received training on animal handling by Scripps DAR and/or Gallay lab staff. For all listed experiments, mice displaying a lack of appetite, slow movement, or other signs of severe discomfort were euthanized immediately upon recommendation of DAR veterinarians and staff. Specific clinical signs included weight loss, hunched body posture, lack of grooming, and dehydration. Mice were sometimes found dead of unknown causes despite not previously showing these signs. Throughout the course of the experiment 19 mice were either euthanized early or found dead out of a total of 119 mice. The remaining 100 animals were sacrificed at the end of the experiment. All animals sacrificed were euthanized by cervical dislocation while under full anesthesia via a nosecone with 2% isoflurane/O_2_. Single-housed animals were kept to a minimum to avoid hypothermia or animal discomfort. However, because all mice in the experiment were male, if a mouse because single-housed due to the death of a cage-mate, it was not possible to transfer it to another cage.

### CCl_4_ model of liver fibrosis

Liver fibrosis was induced in mice using the hepatotoxic agent carbon tetrachloride (CCl_4_) (cat# 270652, Sigma-Aldrich, St. Louis, MO, USA), as previously described [24]. Briefly, 10-week-old male C57BL/6J mice nourished on normal chow and water were given intraperitoneal (IP) injections of 0.2 uL/g CCl_4_ twice weekly for 20 weeks. During each IP injection, mice were briefly anesthetized in an induction chamber using a 2% isofluorane/O_2_ mixture to minimize animal movement and the risk of needle sticks. The CCl_4_ was administered as an 8% CCl_4_/corn oil solution. Therefore, for a typical 20 g mouse, 50 uL of the solution was administered with each IP injection. A fresh 8% CCl_4_/corn oil solution was created each week. Control mice were given identical IP injections of pure corn oil. After 20 weeks, mice were anesthetized, sacrificed by cervical dislocation, and the livers weighed and removed. Livers were dissected into equal halves, one of which was flash frozen in liquid nitrogen and stored at -80°C for later molecular analysis. The other half was suspended in zinc-buffered formalin fixative (cat# 5701ZF, Thermo Fisher Scientific, Waltham, MA, USA) for three days before it could be mounted in a paraffin block for histological analysis.

### Western diet/CCl_4_ model of NAFLD/NASH

Features of the human diseases NAFLD and NASH were reproduced in mice by nourishing them with high-fat, high-sugar, and high-cholesterol western-diet chow and sugar water, together with CCl_4_, as previously described [25]. Briefly, 10-week-old male mice C57BL/6J mice received ad libitum western-diet chow containing 21.1% fat, 41% sucrose, and 1.25% cholesterol by weight (cat# TD.120528, Envigo Teklad, Madison, WI, USA), in lieu of normal chow. They simultaneously received ad libitum sugar-water containing 23.1 g/L fructose (cat# F0127, Sigma-Aldrich) and 18.9 g/L glucose (cat# G8270, Sigma-Aldrich), instead of normal water. Sugar water was prepared in advance, first as an autoclaved 10X stock solution, and then as a 1X working solution which was again autoclaved in the water bottles provided by the Scripps Research vivarium. This diet continued for 20 weeks. During this time mice received twice weekly IP injections of 0.2 uL/g CCl_4_, in an 8% CCl_4_/corn oil solution as described above. Control mice received ad libitum normal chow and water with twice-weekly IP injections of pure corn oil. After 20 weeks, mice were anesthetized, sacrificed by cervical dislocation, and the livers weighed and removed. Livers were dissected into equal halves, one of which was flash frozen in liquid nitrogen and stored at -80°C for later molecular analysis. The other half was suspended in zinc-buffered formalin fixative for three days before it could be mounted in a paraffin block for histological analysis.

### Immunoblotting

Mouse liver tissue was homogenized in buffer comprising 150 mM NaCl, 1% Triton X-100, 0.5% Sodium Deoxycholate, 0.1% SDS, 50 mM Tris base, 5 mM EDTA, 1 mM EGTA, 10 mM Sodium Fluoride, 1 mM Sodium Orthovanadate, 1 mM PMSF, and Halt^™^ Protease and Phosphatase Inhibitor Cocktail (cat# 78440, ThermoFisher Scientific, Waltham, MA). Lysates were subjected to centrifugation at 15,000 x g for 15 min at 4°C to pellet any cell debris, and the protein concentration was determined using the BCA Protein Assay Kit (cat# 7780S, Cell Signaling, Danvers, MA). Samples usually comprising 10–30 μg of protein were mixed with Laemmli sample buffer including 2-mercaptoethanol, heated to boiling for 5 min, and subjected to SDS-PAGE on NuPAGE^™^ 4–12% Bis-Tris gels (cat# NP0336, Invitrogen, Waltham, MA) followed by transferring onto PVDF membrane for immunoblotting analysis. Antibodies were raised against CypA as described previously [26, 27]. Additional antibodies were purchased that were raised against CypB (cat# D1V5J, Cell Signaling, Danvers, MA, USA), KDEL (cat# ADI-SPA-827F, Enzo, Farmingdale, NY, USA), or GAPDH (cat#2118S, Cell Signaling).

### PCR arrays

RNA was extracted from LX-2 human hepatic stellate cell cultures using the RNeasy Plus Mini Kit, according to the manufacturer’s instructions (cat# 74136, Qiagen, Hilden, Germany). RNA concentrations were determined using a Nanodrop^®^ ND-1000 UV/Vis spectrophotometer. PCR arrays were performed on cDNA generated using Qiagen RT2 First Strand Kit (cat# 330401, Qiagen, Germantown, MD, USA). RNA used in the arrays came from 8 WT mouse livers and 5 CypB KO mouse livers. RT2 Profiler PCR Arrays for Mouse Fibrosis (cat# PAMM-120Z, Qiagen, Germantown, MD, USA) were used according to the manufacturer’s instructions.

### Histology

Liver tissue was fixed in zinc-buffered formalin as described above. Livers were then rinsed in 70% ethanol before an hour-long 70% ethanol bath, two hour-long 95% ethanol baths, two hour-long 100% ethanol baths, two hour-long xylene baths, and two four-hour-long liquid paraffin baths. Tissues were then placed in molds with more paraffin to create paraffin blocks suitable for sectioning on a Leica RM2125 microtome. Sections were typically 7μm thick and were incubated on glass histology slides overnight in a 60°C oven.

### Picrosirius red staining

Prepared slides were deparaffinized in a series of three xylene washes, three 100% ethanol washes, two 70% ethanol washes, and two water washes. Slides were then stained for ten minutes in Weigert’s Hematoxylin A (cat# 26044–05, Electron Microscopy Sciences, Hatfield, PA, USA) for nuclei, washed with water, stained for one hour with picrosirius red (cat# 26357–02, Electron Microscopy Sciences, Hatfield, PA, USA) for collagen fibrosis, and washed with acetic acid (cat# 10042–05, Electron Microscopy Sciences, Hatfield, PA, USA). Slides were then dehydrated in the ethanol and xylene washes in reverse order and covered with Permount (cat# SP15-500, FisherSci, Waltham, MA, USA) and a coverslip. Slides were imaged at 4X with a slide scanner. Fibrosis was quantified as a percent of total area of a representative 4X magnification field using ImageJ software.

### Hematoxylin and eosin (H&E) staining

Prepared slides were deparaffinized as above. Slides were then stained for ten minutes in Gill’s Hematoxylin (cat# 72511, Thermo Scientific) for nuclei, washed with water, dipped briefly in Eosin (cat# 7111, FisherSci) cytoplasmic stain, and washed in 1% acid alcohol (cat# 26072–01, Electron Microscopy Sciences). Slides were then dehydrated and prepared as above. Slides were imaged at 10X and 20X with a slide scanner.

### TNFα Immuno-staining

Prepared slides were deparaffinized as above. The presence of tumor necrosis factor alpha (TNFα) in the livers was determined using immunohistochemistry. Slides were submerged in pre-heated sodium citrate buffer (pH 6.0) and heated at 98°C for 20 minutes to retrieve antigens. Liver sections were then incubated with hydrogen peroxide, protein blocking, and streptavidin peroxidase solutions at ten minutes each. Following each incubation step, the sections underwent two washing cycles. Each cycle involved five minutes of gentle agitation in a solution of TBS (Tris-buffered saline, pH 7.0) with 0.025% Triton X-100. Liver sections were then incubated with primary and secondary antibodies i.e., rabbit polyclonal anti-TNFα (cat# ab6671, Abcam, USA) and biotinylated goat anti-rabbit (Abcam, USA). To visualize TNFα, DAB (3,3’-Diaminobenszidine) chromogen was used to produce a brown colored substance at the site of the primary antibody. For histopathology, slides were then counterstained with hematoxylin.

### NAFLD/NASH scoring

H&E images were given a NAFLD Activity Score (NAS), including ballooning, inflammation, and steatosis. Our scoring system for NAS is as follows: ballooning: 0 (no changes), 1 (few ballooned cells), and 2 (many prominent balloon cells); for inflammation: 0 (no changes), 1 (minimal infiltration with no major inflammatory foci), 2 (mild with <2 inflammatory clusters), 3 (moderate with 2–4 foci of leukocytes), and 4 (severe infiltration with >4 inflammatory clusters); for steatosis: 0 (<5% steatosis), 1 (5–33%), 2 (33–66%), and 3 (>66% steatosis). Scores for each category were added together and a score greater than 6 was considered indicative of NASH.

### WT and KO mice

Ppib knockout male mice used in this study were generated so that one Ppib allele has had exon 3 globally deleted in all tissues and cell types. Heterozygous Ppib +/- mice were then cross-bred to generate homozygous Ppib -/- mice which have a complete absence of any CypB protein but without affecting the other cyclophilin family members. Ppib +/- heterozygous knockout mice were a gift of Dr. Richard Bram and were previously described [28]. Ppia knockout male mice used in this study were purchased from Jackson Labs (cat# 005320, Bar Harbor, ME, USA) and were described previously [12]. Both knockout lines were backcrossed with C57BL/6J mice for 10 generations before the beginning of the experiments and other than the knockout gene, were indistinguishable from WT C57BL/6J mice. Age-matched wild-type male C57BL/6J mice were bred and purchased from the Rodent Breeding Colony at Scripps Research. CypA KO and CypB KO experiments were run with their own WT controls to enable greater flexibility, to improve scientific rigor, and to ensure that CypA KO and CypB KO mice were the same age at the start of their respective experiments. PCR primers used for genotyping are as follows:

Ppia—Fwd—5’-CTTTGCAGACGCCACTGTC-3’
Ppia—Rev—5’-CAAAATGGCCCCTCGTCA- 3’
Ppib—Fwd—5’-CTGCTAGGACAAACACTGCT-3’
Ppib—Rev1—5’-CCCACAGTGCTTTCTGGTAT- 3’
Ppib—Rev 2—5’-GGCTCAAACTCAGAGATCCA-3’

### Statistics

All error bars shown are ± standard error of the mean and statistical treatments are generated by unpaired student’s t test when comparing two values or one-way analysis of variance (ANOVA) with Newman–Keuls post hoc analysis when comparing more than two values. Statistics were generated using Graphpad Prism software. Raw data tables are provided in S1–S4 Tables.

## Results

### CypB KO mice, but not CypA KO mice, are protected from CCl_4_-induced liver fibrosis

**Fig 1:**
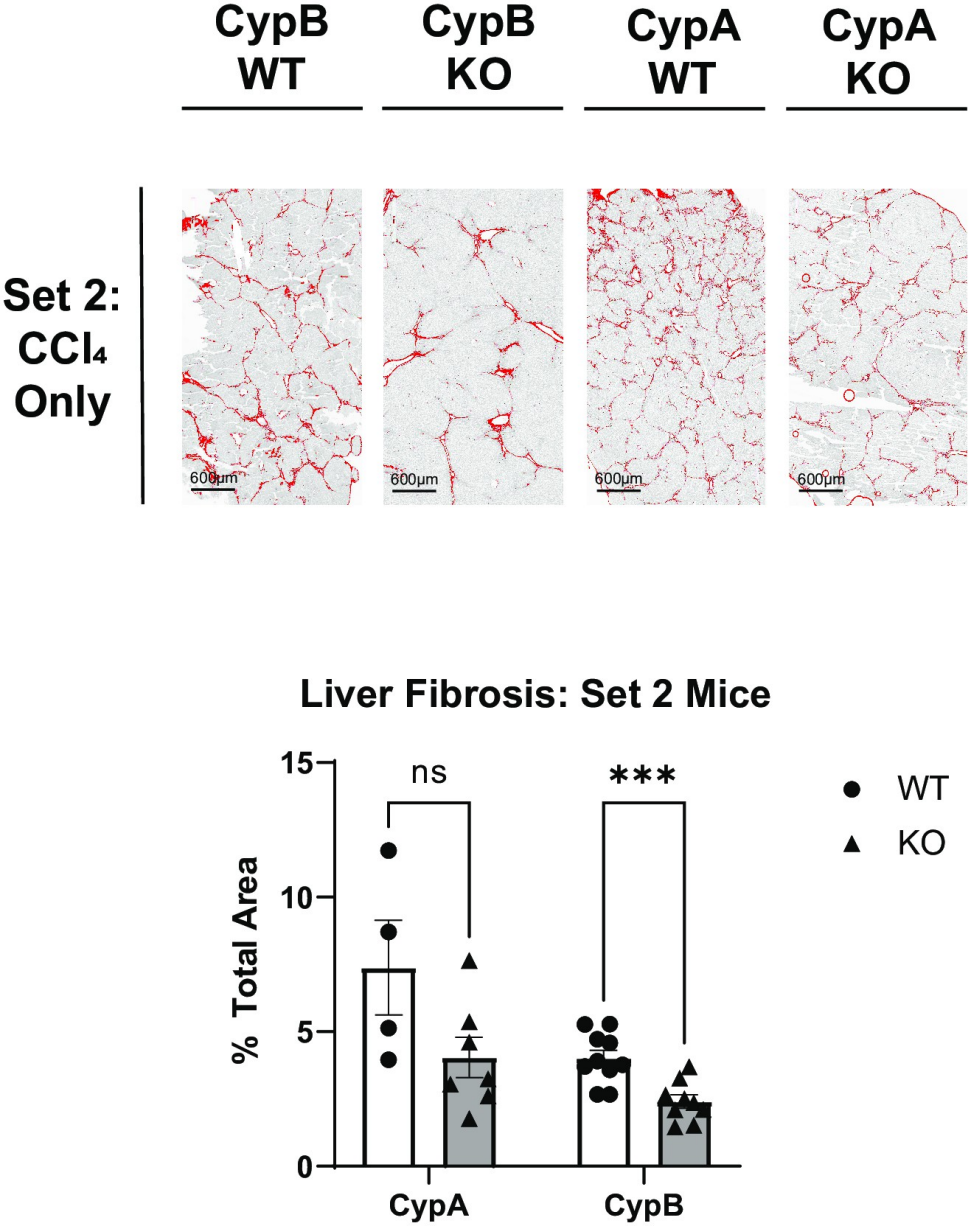
CypB KO mice and not CypA KO mice develop less liver fibrosis after administration of CCl_4_. WT, CypB KO, and CypA KO mice were administered CCl_4_ twice weekly via intraperitoneal (IP) injection for twenty weeks. WT and CypA mice developed significant interlobular branching fibrosis, as determined by histological analysis of picrosirius red-stained liver tissue sections. CypB KO mice however, displayed significantly less stained area relative to other groups. The area of stained tissue relative to the total area was quantified with ImageJ software over several fields per sample. Representative images are shown here. *** p≤0.001 significance between a condition and WT control by unpaired students t-test.

We previously showed that broad inhibition of cyclophilins, through the use of multiple inhibitor compounds is beneficial in mouse models of NAFLD/NASH that feature prominent fibrosis [6, 7]. In each case, fibrosis was significantly blunted in the mice receiving the inhibitor compounds via daily oral gavage. As previously noted, CRV431 in particular was shown to be more effective in this regard than another candidate NAFLD drug, OCA, which is a farnesoid X receptor agonist and does not affect cyclophilins. In order to narrow down which cyclophilin family member’s inhibition is most important in producing the observed beneficial effects of compounds like CRV431, we obtained two mouse lines in which either CypA or CypB had been deleted in all tissues. Both lines were paired with separate WT C57BL/6J controls and separated into three sets. Set 1 was maintained on normal water and chow. Set 2 received normal water and chow but received biweekly IP injections of CCl_4_ in corn oil. Set 3 received sugar solution and western diet chow along with the same biweekly IP CCl_4_ injections. Despite the ubiquitous expression of cyclophilins and their involvement in critical processes, none of the Set 1 cyclophilin-deficient mice appeared abnormal or were distinguishable from normal C57BL/6J mice other than significant differences in weight. Naïve Set 1 CypA KO and CypB KO mice weighed significantly less than their WT controls. Liver weights were similarly lower for CypA KO and CypB KO mice (S1 Fig). Neither naïve knockout line had significant liver fibrosis or signs of NAFLD/NASH at the end of the experimental timeframe, as expected (S2 Fig). All three lines tolerated biweekly intraperitoneal (IP) injections as well as did their WT controls. However, it was noted that the KO lines generally had higher rates of mortality relative to WT controls. Previously reported abnormalities at baseline, such as kyphosis or osteoporosis in CypB KO mice [28], were not observed during the timeframe of our experiments. We thus compared these two lines, along with their separate WT control groups, first in a pure fibrosis model featuring twice-weekly IP injections of the hepatotoxic agent carbon tetrachloride (CCl_4_) for twenty weeks. All Set 2 WT controls saw elevated liver fibrosis, as measured by picrosirius red staining, relative to Set 1 mice, as expected. However, no differences in fibrosis were found in Set 2 CypA KO mice relative to their Set 2 WT controls, despite a trend towards less fibrosis in the CypA KO mice. Strikingly, Set 2 CypB KO mice showed significant reductions in liver fibrosis relative to their controls (Fig 1). This result is novel but consistent with previous findings associating CypB with collagen folding in the endoplasmic reticulum/Golgi secretory pathway [19, 29, 30], since picrosirius red staining primarily reveals collagen fibrosis in the extracellular matrix. It can reasonably be posited that without CypB, collagen folding and export is impaired in these mice, even in conditions with increased hepatotoxicity that likely feature increased fibrotic signaling and myofibroblast activity. As expected, none of the Set 2 mice showed significant steatosis in the liver or other NAFLD/NASH markers, reflecting the CCl_4_ model as a pure fibrosis paradigm (S2 Fig).

Mice and extracted livers were weighed after sacrifice for Sets 1, 2, and 3. Ratios were obtained by dividing liver weights by body weights for each mouse. * p≤0.05 and ***p≤0.001 significance between a condition and WT control by unpaired students t-test.

None of the naïve Set 1 or CCl_4_-only Set 2 groups developed significant steatosis, inflammation, or hepatocyte ballooning, as determined by histological analysis of H&E stained liver tissue sections. NAS were not significantly higher for Set 2 mice than non-diseased Set 1 mice. Representative images are shown here. ***p≤0.001 significance between a condition and WT control by unpaired students t-test.

### CypB KO mice, but not CypA KO mice, are protected from multiple features of disease in a complete NAFLD/NASH model

**Fig 2:**
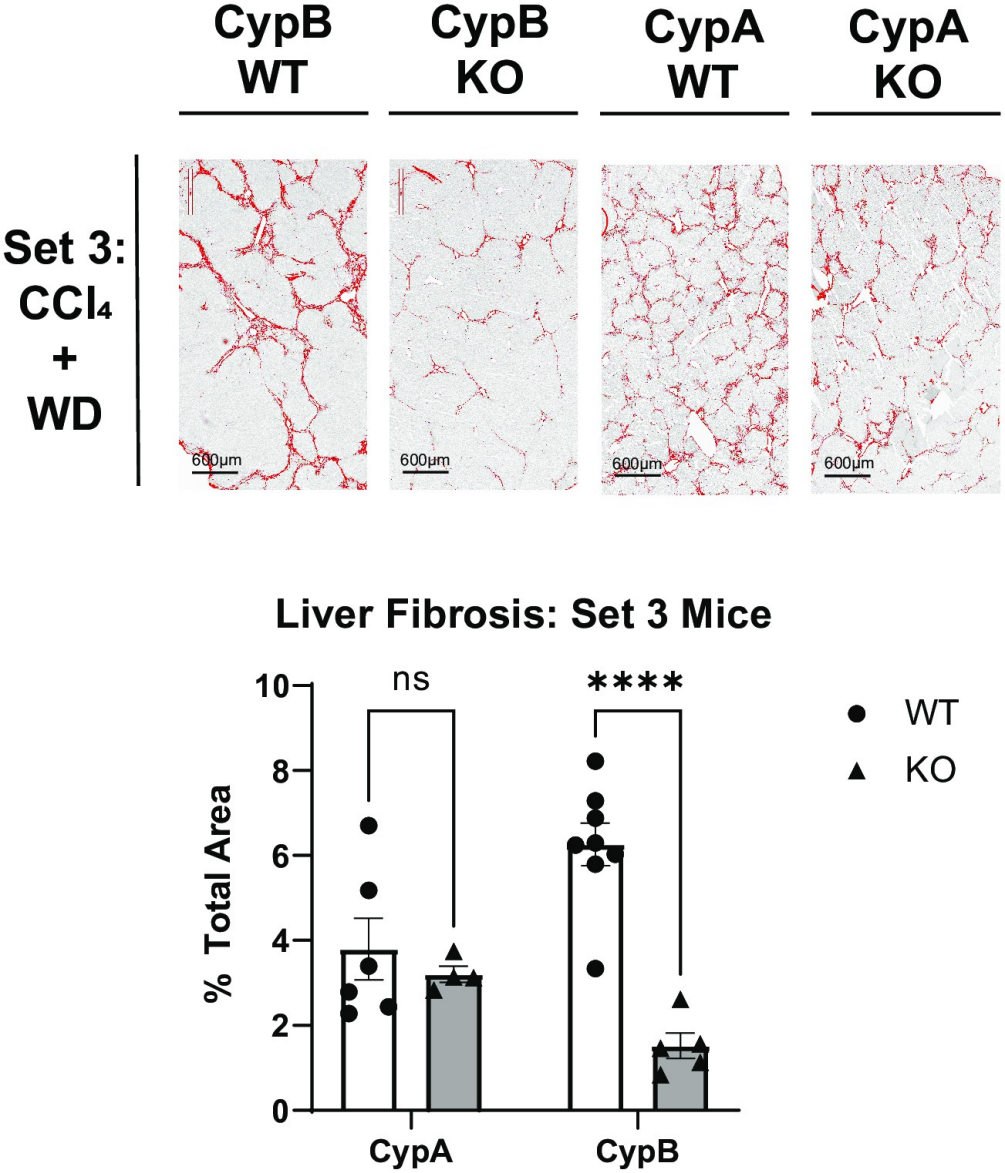
CypB KO mice and not CypA KO mice develop less liver fibrosis after administration of a CCl_4_ and WD model to replicate NAFLD/NASH. WT, CypB KO, CypA KO, and CypD KO mice were nourished with western diet chow and sugar solution while also administered CCl_4_ twice weekly via intraperitoneal (IP) injection for twenty weeks. WT and CypA mice developed significant interlobular branching fibrosis, as determined by histological analysis of picrosirius red-stained liver tissue sections. CypB KO mice however, displayed significantly less stained area relative to WT. The area of stained tissue relative to the total area was quantified with ImageJ software over several fields per sample. Representative images are shown here. ***p≤0.001 significance between a condition and WT control by unpaired students t-test.

**Fig 3:**
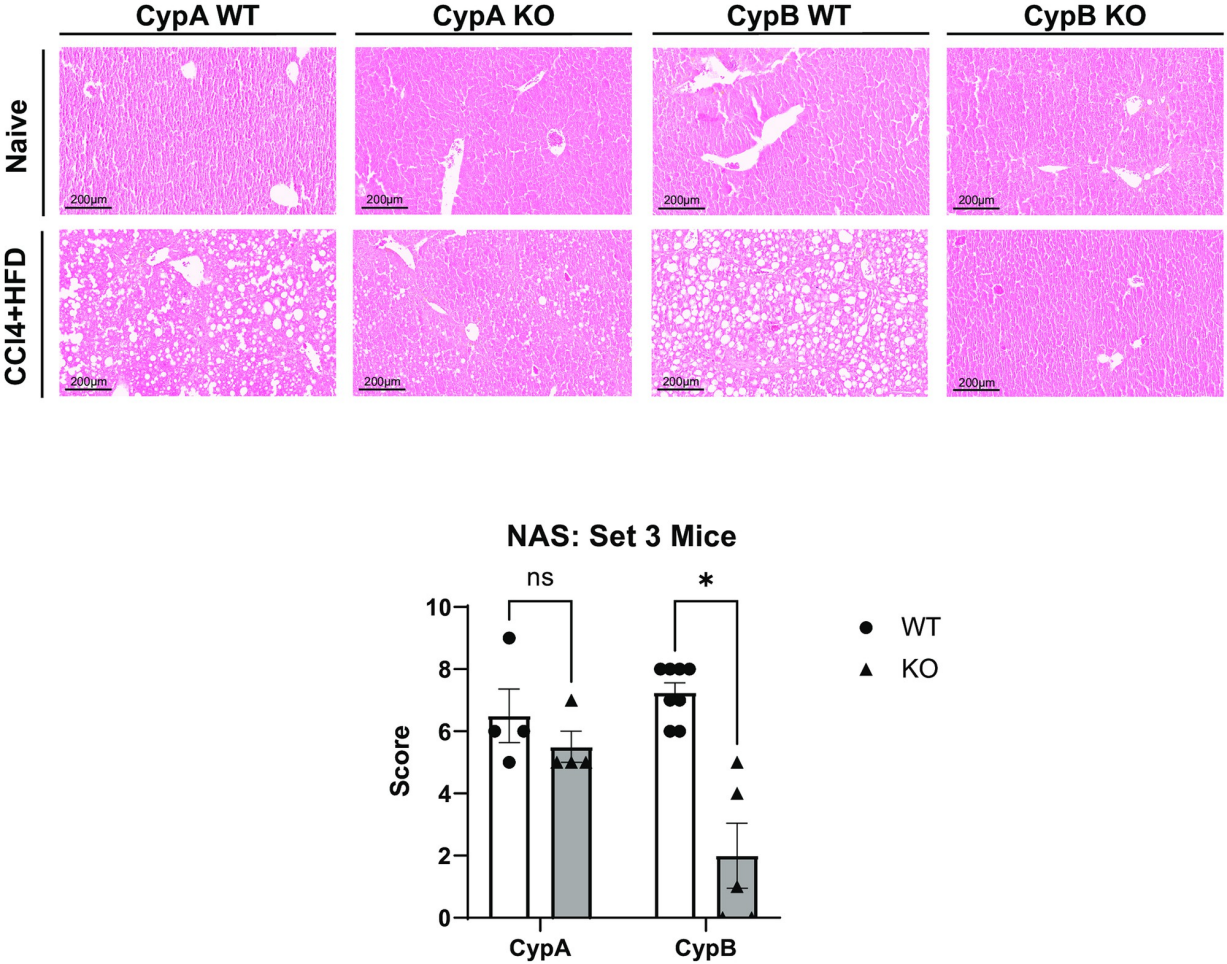
CypB KO mice and not CypA KO mice do not develop essential features of NAFLD/NASH after administration of a CCl_4_ and WD model to replicate NAFLD/NASH. WT, CypB KO, and CypA KO mice were nourished with western diet chow and sugar solution while also administered CCl_4_ twice weekly via intraperitoneal (IP) injection for twenty weeks. WT and CypA mice developed significant steatosis, inflammation, and hepatocyte ballooning, as determined by histological analysis of H&E stained liver tissue sections. NAS were significantly higher for these groups than non-diseased mice. CypB KO mice however, displayed significantly lower NAS relative to WT. Representative images are shown here. ***p≤0.001 significance between a condition and WT control by unpaired students t-test.

**Fig 4:**
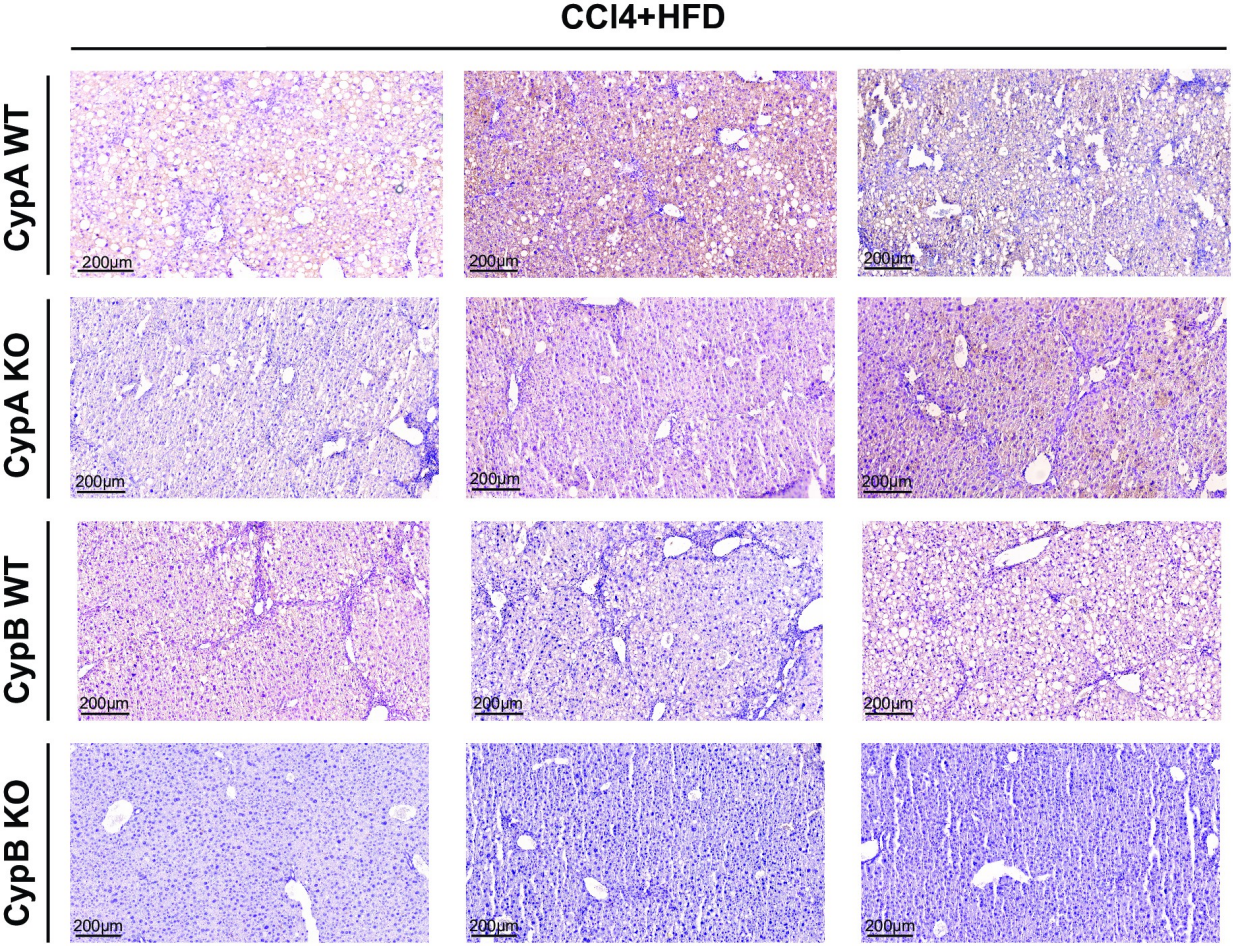
CypB KO livers, but not CypA KO livers, show clearly less inflammatory TNFα staining relative to WT controls after administration of a CCl_4_ and WD model to replicate NAFLD/NASH. WT, CypB KO, and CypA KO mice were nourished with western diet chow and sugar solution while also administered CCl_4_ twice weekly via intraperitoneal (IP) injection for twenty weeks. WT and CypA mice exhibited extensive staining for the inflammatory cytokine TNFα as determined by immunohistochemical staining of liver tissue sections. CypB KO mice however, displayed qualitatively less TNFα staining relative to WT. Representative images are shown here. Blue staining reflects nuclei stained with hematoxylin. Brown coloration from the DAB chromagen reflects areas of reactivity with secondary antibody and thus the presence of TNFα protein. DAB color intensity was not quantified because there is not a linear relationship between antigen concentration and absorbance.

While CCl_4_ alone is useful in inducing acute liver fibrosis, thus elucidating the pathways critical for that one process, it does not encompass many of the characteristics of human liver disease, particularly those of NAFLD/NASH. Combining CCl_4_ with a “western diet” of high fat, high cholesterol, and high sugar chow, along with sugar solution in place of water (CCl_4_+WD), has been shown to reproduce many of the pathological features of human NAFLD/NASH, both at the morphological and transcriptional levels [25]. In order to better understand the roles of individual cyclophilin family members in a mouse model more closely replicating NAFLD/NASH in humans, we subjected a third set of the same three cyclophilin knockout mouse lines to the CCl_4_+WD model for twenty weeks, together with separate WT controls. Following the conclusion of the experiment, fixed mouse liver sections were stained with H&E and were then assessed for signs of NAFLD/NASH via histological scoring for three classic markers of the disease: steatosis, lobular inflammation, and hepatocyte ballooning. Scoring criteria are summarized in Table 1. The three scores are then added together into a NAFLD Activity Score (NAS). Typically, an NAS of 5 or above is predictive of NAFLD/NASH in humans [31]. Set 3 control mice under the CCl_4_+WD regime developed strong signs of NAFLD/NASH with a NAS above 5 as expected. Liver fibrosis was also significantly increased relative to Set 1. While body weight did not increase relative to naïve mice as might have been expected due to the western diet, this was likely due to the frequent CCl_4_ injections which resulted in lower body weights in Set 2 as well (S1 Fig). Set 3 CypA KO mice had a high NAS not significantly different from their controls. Liver fibrosis was also elevated in Set 3 CypA KO mice, as in Set 2. Set 3 CypB KO mice again had reduced fibrosis as in Set 2 (Fig 2). Again, this underlines that CypB, more than the other cyclophilin family members examined, plays a critical role in the development of liver fibrosis under conditions mimicking human NAFLD/NASH. Strikingly, Set 3 CypB KO mice also showed markedly and significantly blunted development of the classic NAFLD/NASH features. All three NAS components were reduced in the CypB KO relative to Set 3 WT mice, and overall NAS was much lower than scores indicative of fully developed NAFLD/NASH. In fact, in terms of NAS Set 3 CypB KO mice were not significantly different than Set 1 non-diseased mice (Fig 3). Additionally, immuno-staining for tumor necrosis factor-alpha (TNFα), an extensively characterized marker of liver inflammation and driver of NAFLD [32–34], showed Set 3 CypB KO livers had qualitatively less staining than their Set 3 WT counterparts, while CypA KO livers had similar staining to their WT controls (Fig 4). Comparing the data from both cyclophilin KO mouse lines relative to their respective controls, it is clear that only mice deficient in CypB had improved outcomes in terms of the most commonly used criteria for diagnosing NAFLD/NASH in humans.

**Table 1:** NASH scoring criteria.

Score	Steatosis	Hepatocyte Ballooning	Lobular Inflammation
0	<5% total area	No ballooned cells	No foci
1	5–33% total area	Few ballooned cells	1 focus
2	33–66% total area	Many ballooned cells	2–4 foci
3	>66% total area	Many ballooned cells	>4 foci

### CypB KO mice have numerous differentially expressed genes related to liver fibrosis and hepatotoxicity relative to WT

**Fig 5:**
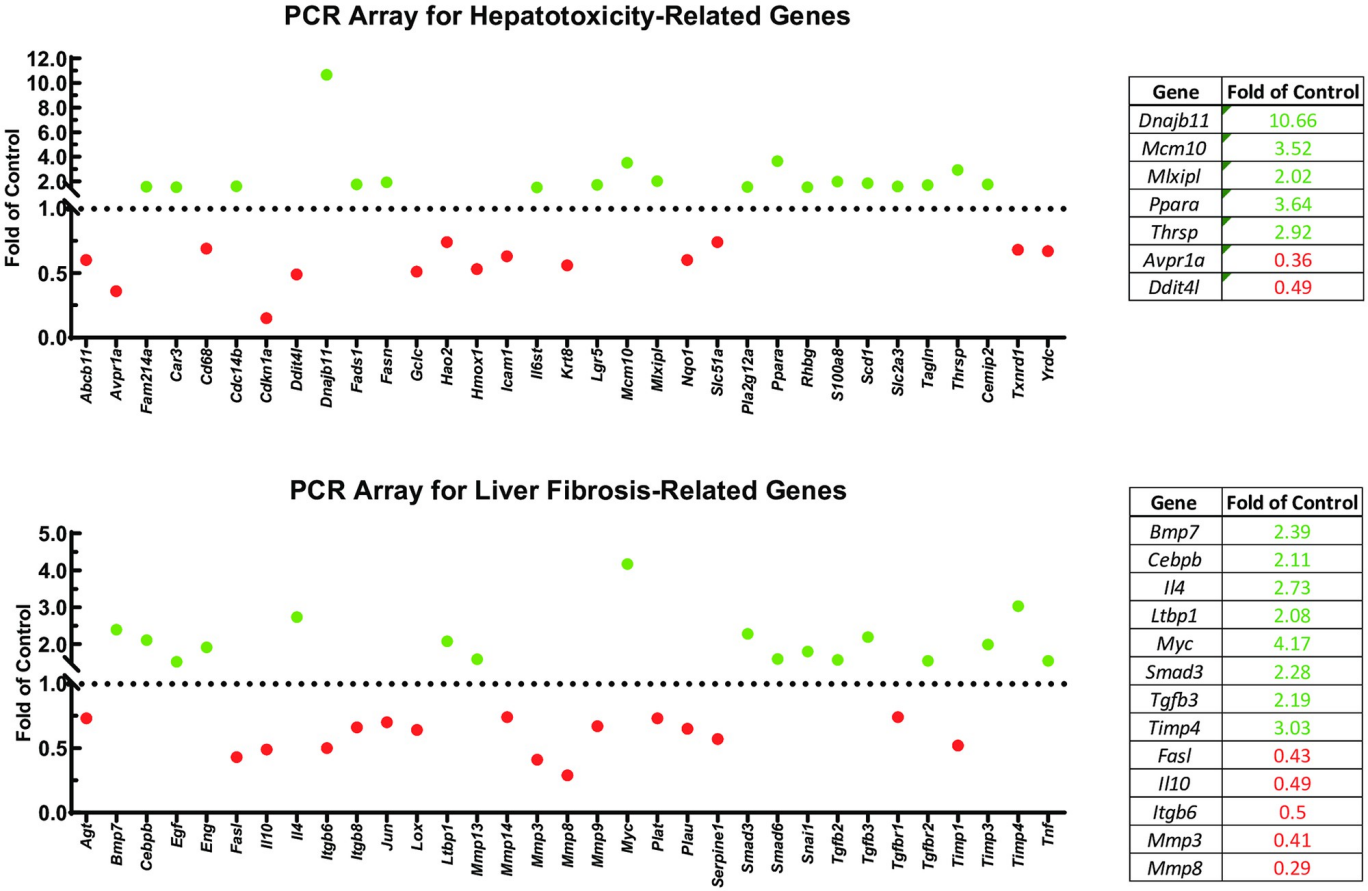
CypB KO mice exhibit differential gene expression compared to WT mice undergoing a NAFLD/NASH model. WT and CypB KO mice were nourished with western diet chow and sugar solution while also administered CCl_4_ twice weekly via intraperitoneal (IP) injection for twenty weeks. RNA isolated from the 8 WT livers and 5 CypB KO livers were analyzed in PCR arrays for genes relevant to hepatotoxicity or liver fibrosis. Green dots represent gene transcripts induced in Set 3 CypB KO mouse livers, while red dots represent decreased expression. Notably induced genes are summarized in the accompanying tables.

To investigate the effect of CypB on the expression of genes that might be involved in NAFLD/NASH disease progression, we performed PCR arrays comparing RNA transcripts isolated from the livers of Set 3 CypB KO or WT mice. Consistent with the results above, we found that numerous fibrosis- or hepatotoxicity-related genes were differentially expressed in the CypB KO mice (Fig 5). Notable differentially regulated genes are also summarized in Fig 5. The gene with the highest induction in either array was *Dnajb11* encoding the protein ERdj3, a protein chaperone associated with endoplasmic reticulum-associated degradation (ERAD), the degradation of terminally misfolded proteins in the ER. While immunoblotting of commonly expressed ER stress effectors did not show elevated ER stress in CypB KO mice (S3 Fig), the possibility that specific actors like ERdj3 are compensating for the lack of CypB should be further investigated in the future.

Liver extracts from Set 3 WT or Set 3 CypB KO or Set 3 CypA KO mice were probed with KDEL antibodies to determine the activation status of the ER stress response. No difference was observed. The same membrane was also probed with CypB or CypA antibodies respectively as well as a GAPDH housekeeping standard.

## Discussion

This study shows the importance of CypB in the progression of fatty liver disease, particularly in the aspects of fibrosis and the defining features of NAFLD/NASH. Without CypB, mice did not develop significant liver fibrosis, even after 20 weeks of bi-weekly IP injections of the hepatotoxic agent CCl_4_. Likewise, fibrosis and NAFLD/NASH was significantly diminished in CypB deficient mice in a more complete model of the disease which included western diet. Since western diet alone does not produce all features of NAFLD/NASH in mice under this timeframe, CCl_4_ injections were maintained as a fibrosis accelerant. Previous studies produced by the Gallay group showed that pan-cyclophilin inhibition, whether from the cyclosporin A-derivative CRV431 or the sanglifehrin-derivative NV-556, had similar effects under comparable disease models in mice [6, 7]. This study thus strongly suggests that the mechanism behind those results was the inhibition specifically of CypB, and not CypA.

We initially theorized that the *in vivo* results for CypB KO mice would also be due to impaired hepatic stellate cell activation, since that cell population is most responsible for fibrotic deposition in the liver. However, transcript levels of HSC activation markers were not decreased in Set 2 or Set 3 CypB KO livers at the end of twenty weeks (not shown). Furthermore, in a PCR panel of select fibrosis-related genes, a majority were found to be elevated in Set 3 CypB KO livers, suggesting that fibrotic signaling was in fact increased despite the significant decrease in actual collagen fibrosis observed (Fig 5). Further studies should confirm whether increases in fibrotic signaling reflect a hyper-activated state of HSCs/myofibroblasts in CypB KO livers, perhaps in an effort to compensate for the lack of collagen deposition.

The role of CypB as a protein folding chaperone localizing to the endoplasmic reticulum implies that another potential mechanism for its observed role in liver fibrosis in mice is that it is specifically important in the folding and export of collagen and perhaps other extracellular matrix components. Mature collagen protein has a complex 3-dimensional shape involving the formation of helical procollagen strands which, are crosslinked together to form a triple-helix. This process involves the activity of multiple enzymes and chaperone protein complexes. There is strong evidence that CypB regulates this process, particularly by affecting hydroxylation of lysine residues, consistent with its role as part of the prolyl-3-hydroxylase complex. The rate of procollagen folding is overall impaired in CypB KO mice, resulting in defects such as osteogenesis imperfecta [19, 28–30, 35].

The importance of CypB and not CypA in the development of other features of NAFLD/NASH, such as steatosis, inflammation, and hepatocyte ballooning, was striking. Given our earlier findings that pan-cyclophilin inhibition via CRV431 or NV556 both reduced fibrosis and HCC development, but not necessarily other characteristics of NAFLD/NASH such as steatosis or inflammation, this finding was unexpected, but likely reflects differences in the loss of function models we tested. Global knockout CypB mice are deficient in CypB throughout development and thus may have differential responses to insults than mice given acute drug treatments as adults. It should also be noted that because of extensive backcrossing into C57BL/6 mice, the knockout mouse lines are 99.9% genetically identical to the WT C57BL/6 control mice and thus the only detectable difference is the knockout. The initiation and progression of NAFLD/NASH characteristics is still not fully understood, but likely reflects imbalances between the rate of fatty acid uptake by the liver, denovo lipogenesis in the liver, and the rate of fatty acid metabolism in the liver. Further research will be needed to determine whether CypB plays a role in any or all of these axes and if so, in what tissue(s) it is most critical.

## Supporting information

S1 Fig Body and liver weights of CypA and CypB KO mice were reduced relative to WT controls while liver to body weight ratios were mostly unchanged. (TIF)

S2 Fig Neither WT nor CypA or CypB KO Set 1 or Set 2 mice showed significant increases in NAS scoring. (TIF)

S3 Fig Set 3 CypB KO mice did not exhibit chronically activated ER stress markers relative to Set 3 WT mice. (TIF)

S1 Table CypA WT mice measurements and data. Raw data from Set 1, 2 and 3 CypA WT control mice were taken immediately after sacrifice. Histological data are also included. Averages, standard deviation, and standard error are listed for each set.
(TIF)

S2 Table CypA KO mice measurements and data. Raw data from Set 1, 2 and 3 CypA KO mice were taken immediately after sacrifice.Histological data are also included. Averages, standard deviation, and standard error are listed for each set.
(TIF)

S3 Table CypB WT mice measurements and data. Raw data from Set 1, 2 and 3 CypB WT control mice were taken immediately after sacrifice. Histological data are also included. Averages, standard deviation, and standard error are listed for each set.
(TIF)

S4 Table CypB KO mice measurements and data. Raw data from Set 1, 2 and 3 CypB KO mice were taken immediately after sacrifice. Histological data are also included. Averages, standard deviation, and standard error are listed for each set.
(TIF)
