# Supplementary figures and images for: Mice lacking cyclophilin B, but not cyclophilin A, are protected from the development of NASH in a diet and chemical-induced model
Source: PLoS One. 2024 Mar 1;19(3):e0298211. doi: 10.1371/journal.pone.0298211 (PMC10906846; doi:10.1371/journal.pone.0298211)

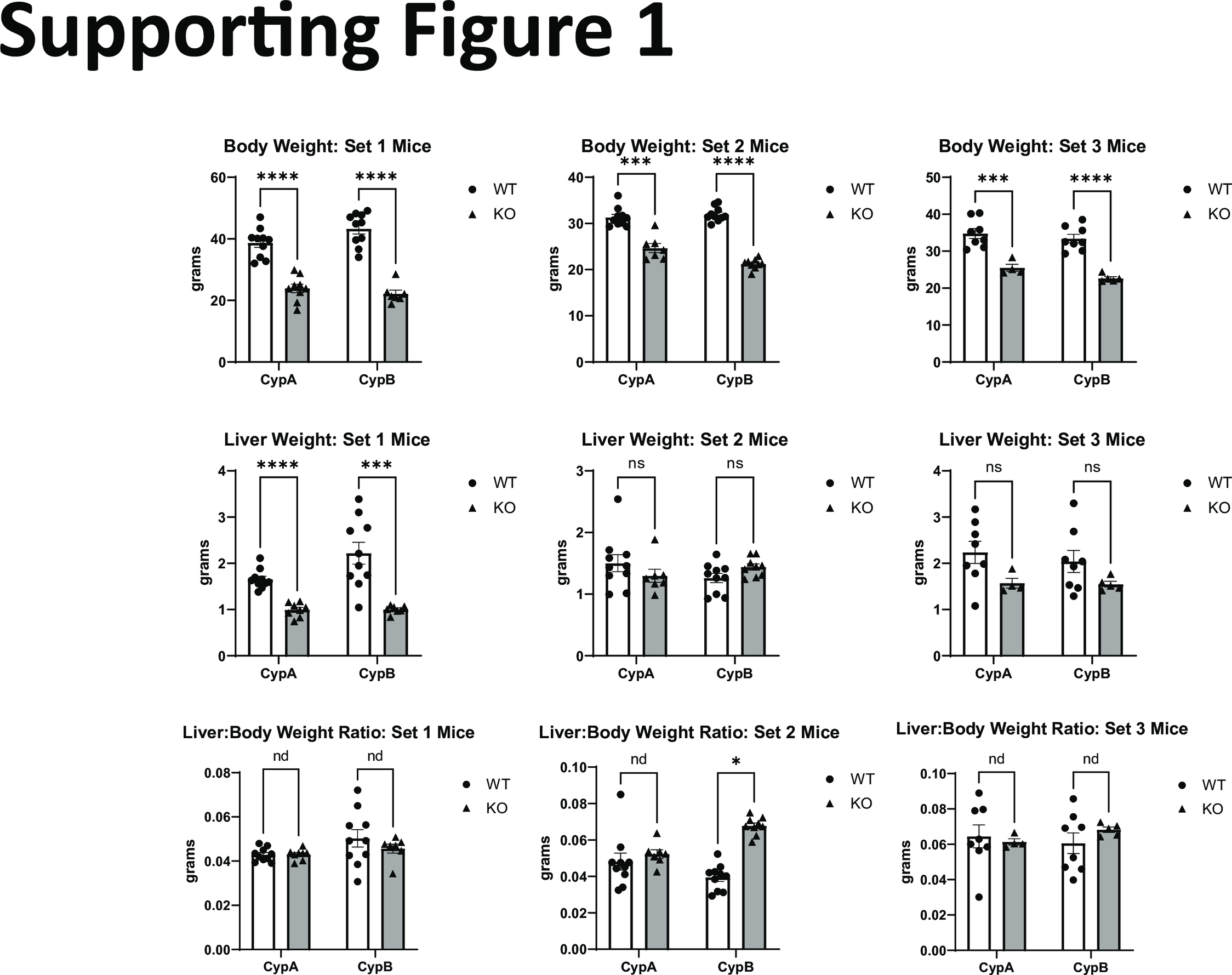

Supplement: S1 Fig — (TIF) [file pone.0298211.s001.tif]

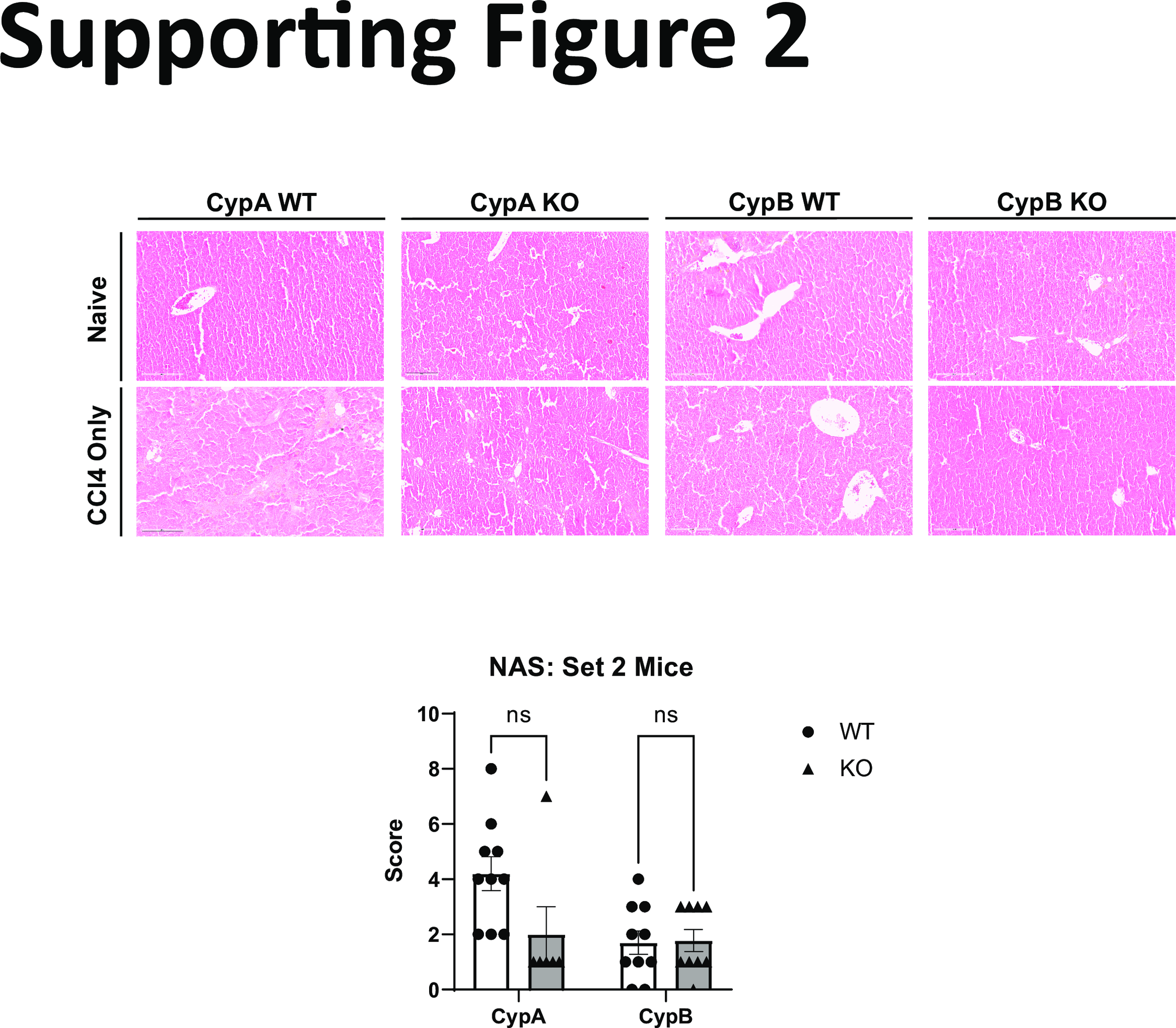

Supplement: S2 Fig — (TIF) [file pone.0298211.s002.tif]

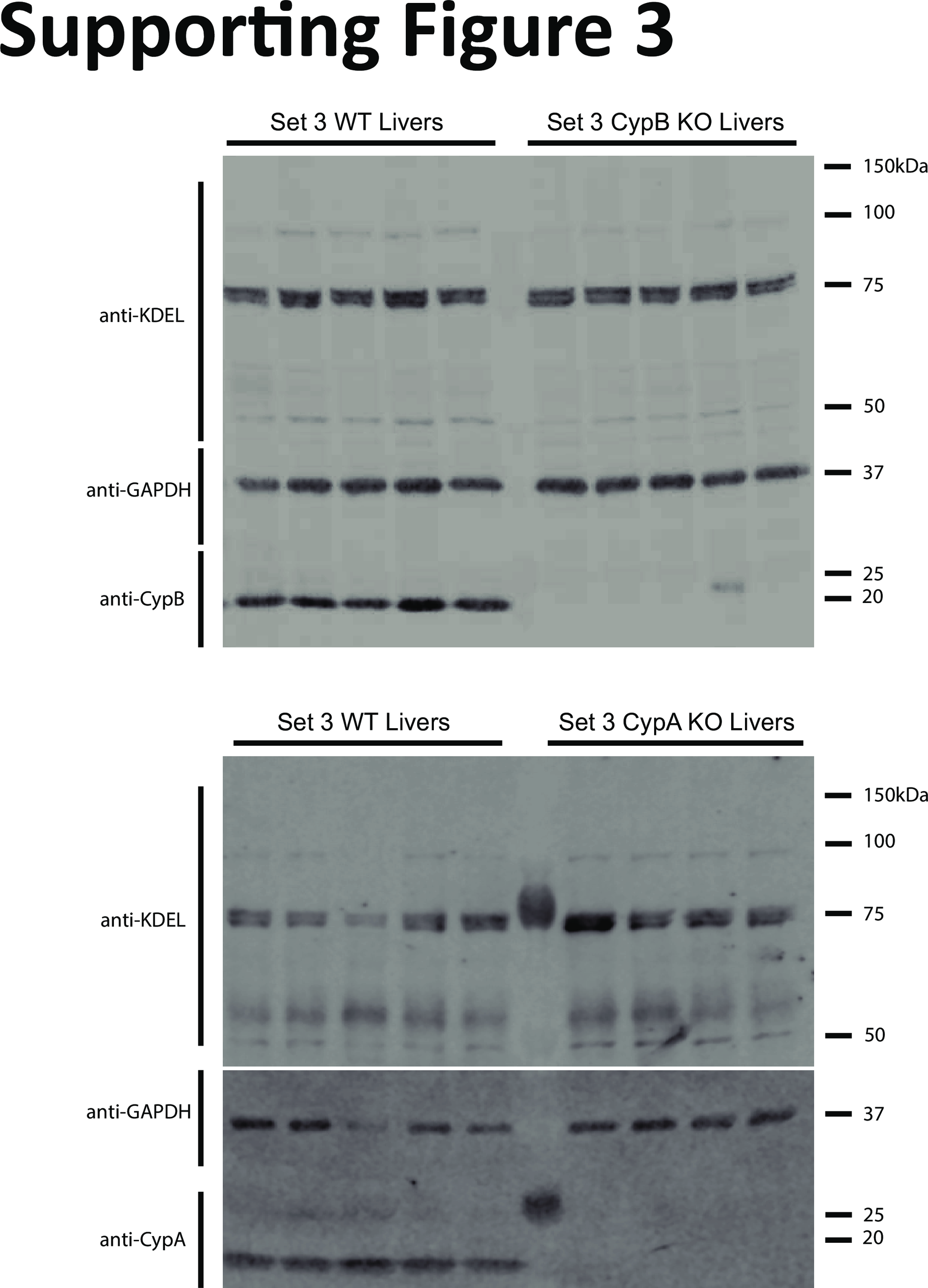

Supplement: S3 Fig — (TIF) [file pone.0298211.s003.tif]

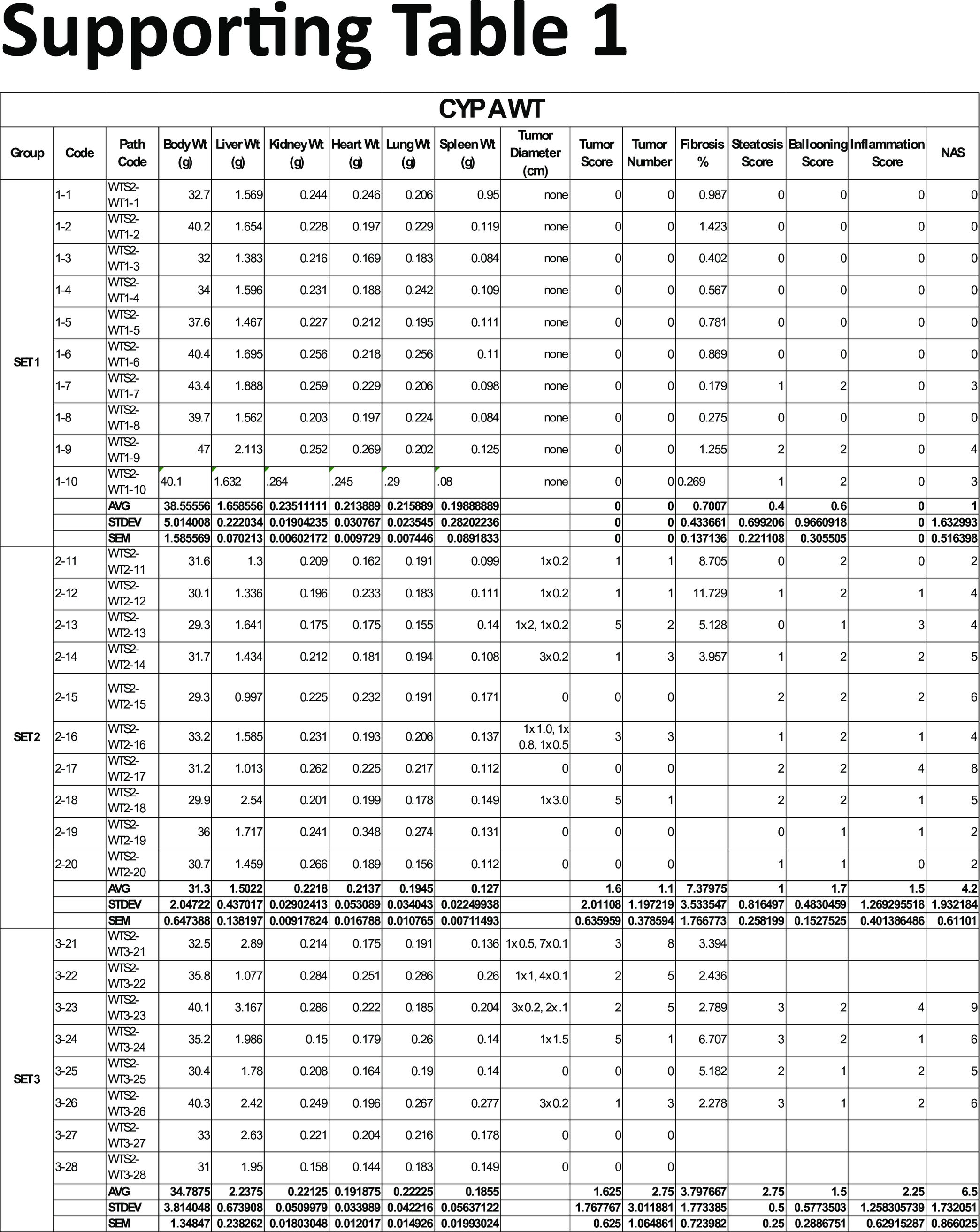

Supplement: S1 Table — Raw data from Set 1, 2 and 3 CypA WT control mice were taken immediately after sacrifice. Histological data are also included. Averages, standard deviation, and standard error are listed for each set. (TIF) [file pone.0298211.s004.tif]

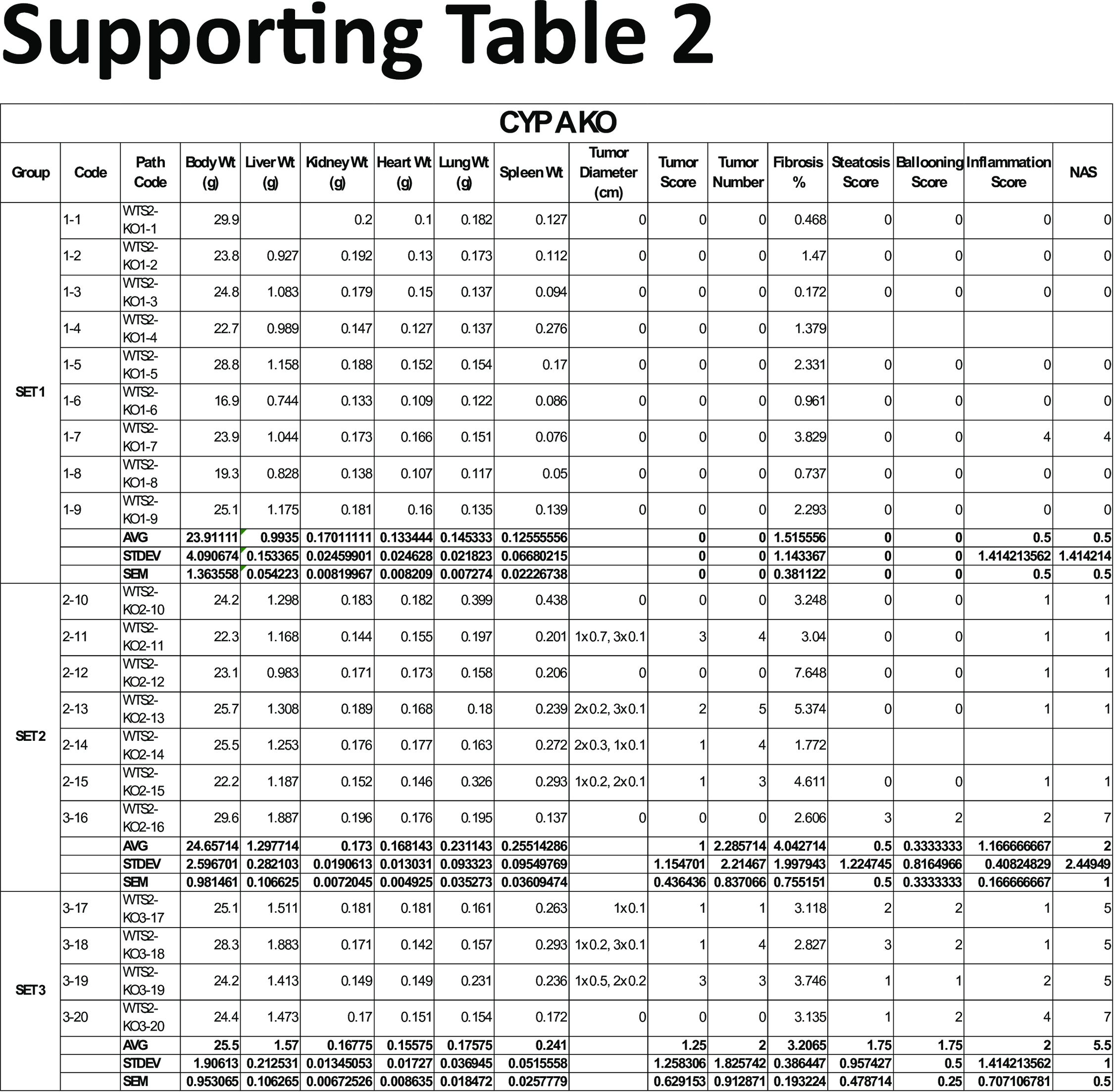

Supplement: S2 Table — Raw data from Set 1, 2 and 3 CypA KO mice were taken immediately after sacrifice.Histological data are also included. Averages, standard deviation, and standard error are listed for each set. (TIF) [file pone.0298211.s005.tif]

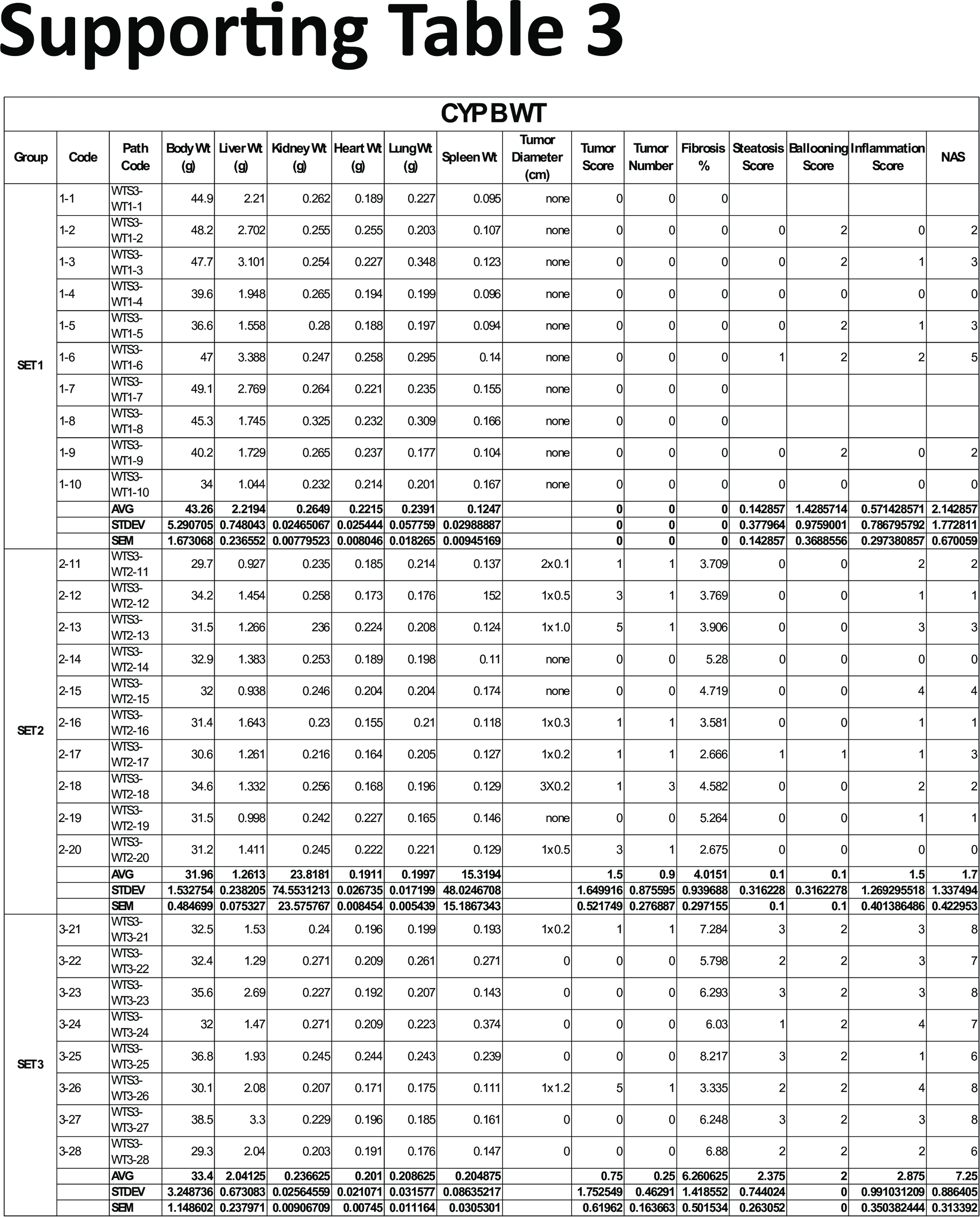

Supplement: S3 Table — Raw data from Set 1, 2 and 3 CypB WT control mice were taken immediately after sacrifice. Histological data are also included. Averages, standard deviation, and standard error are listed for each set. (TIF) [file pone.0298211.s006.tif]

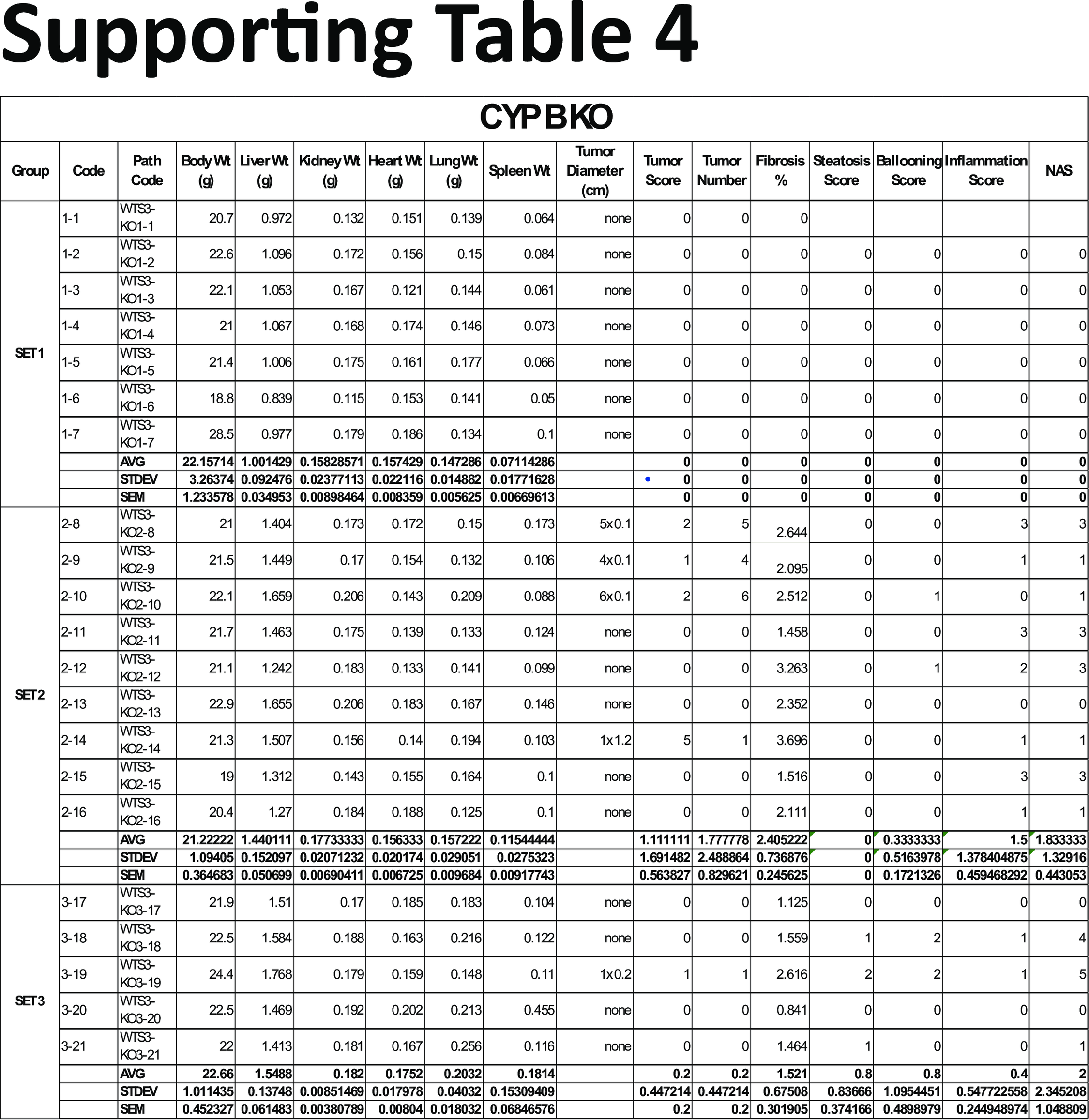

Supplement: S4 Table — Raw data from Set 1, 2 and 3 CypB KO mice were taken immediately after sacrifice. Histological data are also included. Averages, standard deviation, and standard error are listed for each set. (TIF) [file pone.0298211.s007.tif]
